# An unusual presentation of Huntington’s disease

**DOI:** 10.1002/ccr3.4547

**Published:** 2021-07-16

**Authors:** Martin Schulze Westhoff, Alma Osmanovic, Catharina Meissner, Johannes Heck, Nima Mahmoudi, Corinna Hendrich, Georg Berding, Johanna Seifert, Stefan Bleich, Helge Frieling, Tillmann Krüger, Adrian Groh

**Affiliations:** ^1^ Department of Psychiatry Social Psychiatry and Psychotherapy Hannover Medical School Hannover Germany; ^2^ Department of Neurology Hannover Medical School Hannover Germany; ^3^ Institute for Clinical Pharmacology Hannover Medical School Hannover Germany; ^4^ Department of Diagnostic and Interventional Neuroradiology Hannover Medical School Hannover Germany; ^5^ Institute for Human Genetics Hannover Medical School Hannover Germany; ^6^ Department of Nuclear Medicine Hannover Medical School Hannover Germany

**Keywords:** 18F‐FDG PET/CT, frontotemporal dementia, Huntington's disease, psychosis, tauopathies

## Abstract

We describe the case of a 59‐year‐old woman who exhibited psychotic symptoms, cognitive dysfunction, and restlessness. While the clinical picture and 18F‐FDG PET/CT suggested the presence of a tauopathy, especially frontotemporal dementia or progressive supranuclear palsy, genetic testing eventually revealed Huntington's disease.

## Graphical abstract

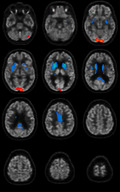

## 1 INTRODUCTION

Huntington's disease (HD) is an autosomal dominantly inherited neurodegenerative disease characterized by motor dysfunction, cognitive impairment, and psychopathological alterations.^1^ HD is caused by an expansion of the trinucleotide cytosine‐adenine‐guanine (CAG) repeat in the huntingtin (*HTT*) gene located on chromosome 4, which results in the production of a toxic variant of the eponymous protein huntingtin, leading to polyglutamine aggregates and subsequently to neuronal dysfunction and cell death, particularly in the striatum.^2^, ^3^ Hyperkinetic movement disturbances with prominent chorea are typically present in early stages of the disease.^1^, ^4^ As HD progresses, other motor deficits become more evident, culminating in dystonia or tics accompanied with rigidity, bradykinesia, and postural instability.^5^ Furthermore, cognitive decline can manifest many years before the onset of motor symptoms, presenting as impairment of executive functions, visuospatial perception, and short‐term memory.^1^ Behavioral changes in HD can mimic the behavioral variant of frontotemporal dementia (bv‐FTD).^6^ A partial overlap between HD and different subtypes of dementia has been described in the medical literature since elevated levels of tau protein in cerebrospinal fluid (CSF) and intraneuronal inclusions with an amyloid‐like structure have been detected in HD patients.^7^, ^8^ Psychopathological alterations such as apathy, disinhibition, irritability, and depressive symptoms are well‐known disease characteristics of HD.^1^, ^9^ Psychosis, on the other hand, is less frequent.^9^, ^10^, ^11^ Psychosis in the context of HD is an ill‐defined term, most frequently referring to hallucinations and delusions.^10^ To date, only symptomatic pharmacological options such as vesicular monoamine transporter inhibitors like tetrabenazine, antidepressants, and antipsychotics are available for the treatment of HD, making HD a fatal and currently incurable disease with reduced life span.^1^, ^4^ Most HD patients die by the age of 55 years.^1^

## 2 CASE PRESENTATION

A 59‐year‐old woman without previous history of psychiatric or neurological disease admitted herself to the emergency department of our university hospital with existential fears regarding her state of unemployment. Due to massive anxiety, a structured conversation with the patient was only feasible after anxiolytic treatment with lorazepam. During the clinical examination, it became apparent that the patient's fears were elicited by delusional ideas. The patient claimed that thoughts had been inserted into her head and were being controlled by someone else. Moreover, she reported ideas of audible thoughts. An organic schizophrenia‐like disorder was assumed as the most likely diagnosis, and the patient was transferred from the emergency department to the Department of Psychiatry, Social Psychiatry, and Psychotherapy of our clinic. The patient claimed that her mother had been diagnosed with Alzheimer's disease at the age of 50 years and had died at the age of 65 years. Other family members had not been afflicted with neurological or psychiatric disease.

Laboratory investigations revealed a mild hypothyreosis due to status post hemithyroidectomy; hence, levothyroxine was substituted. Moreover, hyponatremia was identified and sodium chloride tablets were administered. Additionally, the patient was advised to reduce drinking tap water, of which she was consuming up to 4 liters per day. No abnormalities were detected in the complete blood count. Deficiencies of folic acid, vitamin B_12_, and ceruloplasmin as well as infection with human immunodeficiency virus, *Borrelia burgdorferi*, and *Treponema pallidum* could also be excluded.

After completion of the laboratory investigations, we initiated an antipsychotic treatment with risperidone. However, delusional ideas persisted despite treatment with risperidone. Due to sleep disturbances, we commenced a treatment with chlorprothixene, while risperidone was discontinued.

Further neurological examination revealed dysdiadochokinesis (predominantly of the left hand), mild hyperkinetic movements (particularly restlessness), bradykinesia in the finger‐tapping test, and a broad‐based gait. Rigidity, tremor, or postural instability was not present. In the meantime, the patient became increasingly disoriented to time and space and exhibited long‐term memory deficits accompanied by episodes of fear, agitation, and perseverations, indicative of cognitive impairment. Cognitive function was assessed using the Consortium to Establish a Registry on Alzheimer's Disease neuropsychological assessment battery (CERAD+)^12^, ^13^ which detected deficits in recognition of verbal content, a severe impairment of visuospatial abilities, a reduction in semantic and phonological word fluency, and a decreased cognitive flexibility (the latter was additionally evidenced in the Trail Making B test). Furthermore, we performed the Mini‐Mental State Examination (MMST) and the frontal assessment battery (FAB) where the patient scored 22 out of 30 points and 10 out of 18 points, respectively, indicating reductions in mental flexibility, motor programming, environmental autonomy, and inhibitory control.^14^ The Structured Clinical Interview for Diagnostic and Statistical Manual of Mental Disorders IV Axis II Personality Disorders (SCID‐II) showed no evidence of a personality disorder, while participation in the Beck Depression Inventory II (BDI‐II) was refused by the patient.^15^, ^16^ Due to clinically predominant executive dysfunction and the described psychopathological symptoms, we considered bv‐FTD as the most likely diagnosis.

**FIGURE 1:**
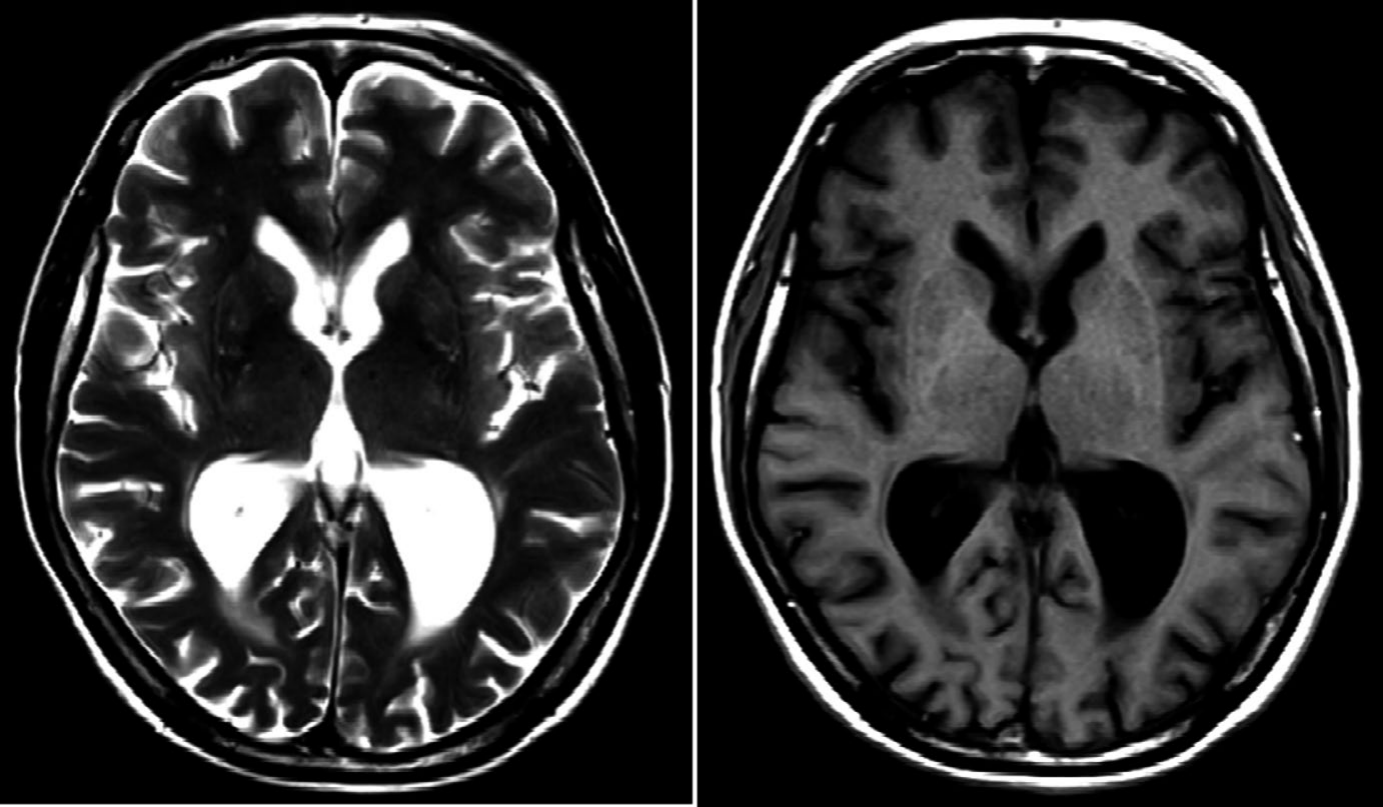
T1‐ and T2‐weighted magnetic resonance imaging exhibiting cerebral atrophy with putaminal accentuation and ventriculomegaly

However, the neurological symptoms displayed by our patient could not sufficiently be explained by bv‐FTD; therefore, an electroencephalogram was performed, which demonstrated a physiological alpha rhythm without signs of epileptiform potentials. Magnetic resonance imaging (MRI) revealed generalized cerebral atrophy with putaminal accentuation (putaminal rim sign), a hyperintensity at the lateral margin of the putamen, and ventriculomegaly (Figure 1). A lumbar puncture with examination of CSF parameters including tau and beta‐amyloid protein was unremarkable. Furthermore, intrathecal immunoglobulin synthesis and the presence of autoimmune encephalitis antibodies and protein 14–3–3 in CSF were excluded. Altogether, we suspected neurodegeneration as the underlying cause of all of the patient's symptoms including movement disturbances, symptoms of dementia, and psychiatric features.

**FIGURE 2:**
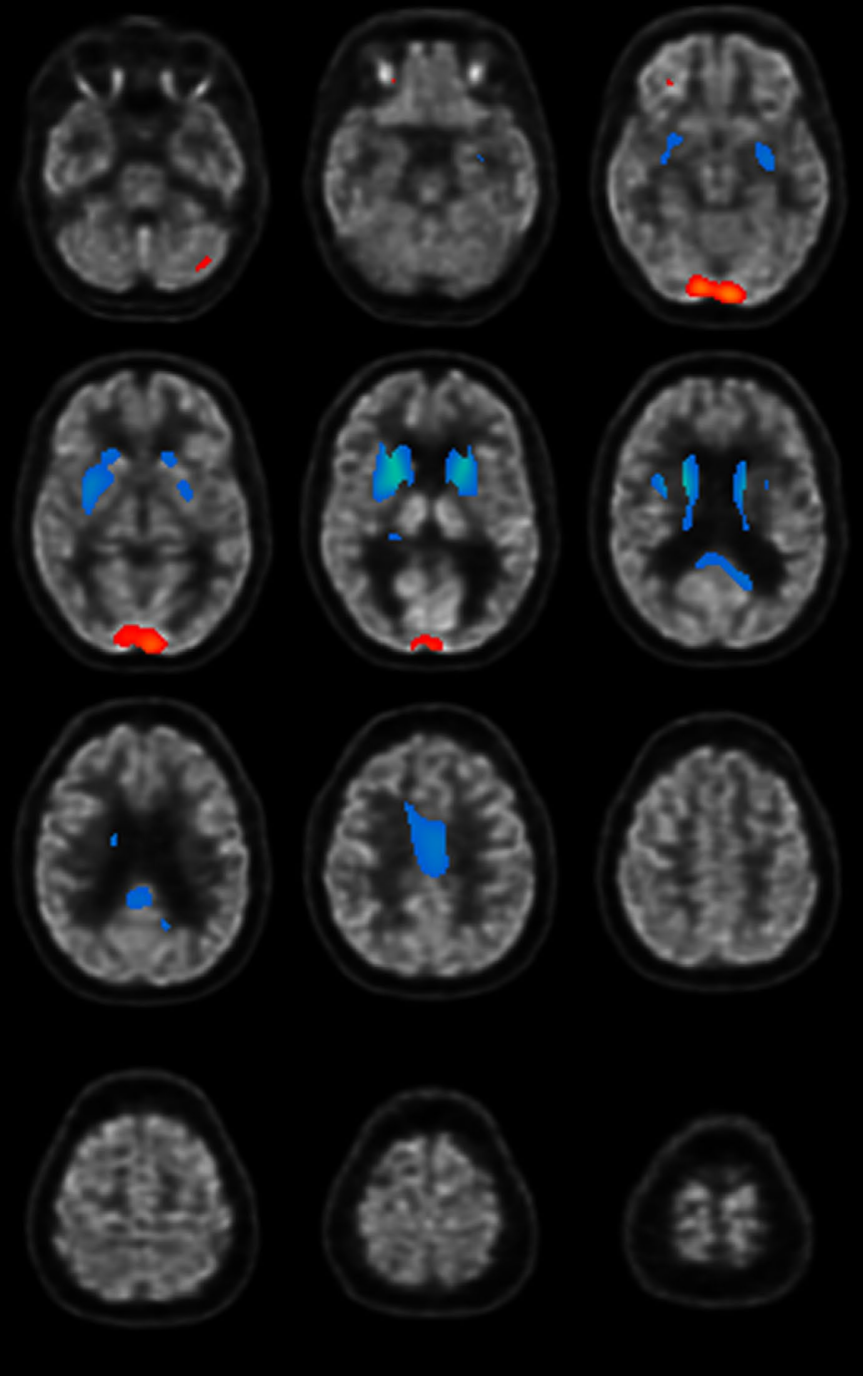
Statistical parametric maps overlaid to 18F‐FDG PET/CT images showing striatal hypometabolism and occipital hypermetabolism

To differentiate neurodegenerative disease patterns, cerebral glucose metabolism was investigated by ^18^F‐fluoro‐2‐deoxy‐D‐glucose positron emission tomography/computed tomography (^18^F‐FDG PET/CT) which revealed global cerebral glucose hypometabolism. Glucose uptake was particularly low in the striatum, frontal inferior cortex (opercular part), cingulum, precuneus, hippocampus, and amygdala when compared to a control group of corresponding age. In contrast, glucose hypermetabolism could be observed in the occipital lobe (Figure 2). Such a glucose metabolism pattern was evaluated unusual in pure dementia and pointed to a neurodegenerative disease with primary involvement of the basal ganglia. Of note, glucose hypometabolism in the striatum has been observed in atypical Parkinson syndromes, especially in progressive supranuclear palsy (PSP), neuroacanthocytosis syndromes, and HD. This consideration led to genetic testing in order to determine the number of CAG repeats in the *HTT* gene. People with fewer than 29 CAG repeats never develop HD, while the presence of 39 or more CAG repeats inevitably leads to the development of HD.^1^, ^3^ In our patient, genetic testing revealed 42 CAG repeats in the *HTT* gene, confirming the diagnosis of HD.

Over the course of inpatient care, the patient required support from the nursing staff for virtually all activities of daily living, including personal hygiene. Moreover, she was severely disoriented in both space and time. Therefore, the patient was assigned a legal guardian. Following discharge from our clinic, the patient was admitted to a residential home for patients with chronic psychiatric and neurological diseases.

## 3 DISCUSSION

We have presented the case of a 59‐year‐old woman displaying psychotic symptoms in conjunction with cognitive decline and behavioral alterations indicative of bv‐FTD. Due to the inability of the presumed diagnosis of bv‐FTD to provide substantial explanation for the patient's unusual clinical manifestation, further diagnostic measures were applied. ^18^F‐FDG PET/CT revealed a distinctive cerebral glucose hypometabolism pattern suggestive of a tauopathy. Genetic testing—finally—revealed the correct diagnosis: Huntington's Disease.

Usually, the first symptoms in HD patients become apparent at the age of about 40 years. Of note, a higher number of CAG repeats causes an earlier onset of clinical symptoms.^1^, ^4^ Our patient was 59 years old when she exhibited symptoms of HD for the first time. It is widely accepted that psychopathological abnormalities including depressive episodes frequently precede the development of neurological symptoms in patients suffering from HD.^17^ Psychotic symptoms in the context of HD are rare and pose a therapeutic challenge as they are often resistant to treatment.^18^, ^19^, ^20^

To the best of our knowledge, this is the first report describing symptoms of delusion of reference and feelings of mind manipulation in a patient with HD. Data about psychosis in the context of HD are sparse and inconsistent; they do, however, link psychotic symptoms to a younger age at first manifestation, cognitive impairment, and a lower number of CAG repeats in the *HTT* gene.^10^ Consequently, the psychotic symptoms presented by our patient may be considered unusual for HD.

There are reports in the literature describing bv‐FTD with dyskinesia similar to motor symptoms in HD, in which HD could be excluded by genetic testing.^21^ Conversely, a case report of a patient with behavioral changes and executive dysfunction similar to bv‐FTD, but eventually diagnosed with HD, is available.^6^

Our case demonstrates the enormous value of neuroimaging techniques in patients presenting with a psychotic‐dysexecutive syndrome accompanied by symptoms of a movement disorder. Whereas MRI showed global brain atrophy (a rather unspecific finding), ^18^F‐FDG PET/CT revealed a unique cerebral glucose metabolism pattern and contributed significantly to the diagnosis of HD. A loss of volume and glucose hypometabolism is commonly seen in the striatum and sometimes in several other brain regions in HD.^22^ We detected glucose hypometabolism not only in the striatum, but also in the inferior frontal cortex (opercular part), precuneus, amygdala, cingulum, and hippocampus. Indeed, lower metabolism particularly in frontotemporal and parietal cortices has been linked to disease progression as evident in the presented case.^23^ Furthermore, glucose hypermetabolism was observed in the occipital lobe. In the context of HD, glucose hypermetabolism has only rarely been reported. Its role with regard to pathogenesis remains unresolved.^23^ Nevertheless, in the presented case, hypermetabolism in the occipital lobe may not be a distinct disease characteristic of HD, but may instead represent an artifact elicited by visual activity. The unusual cerebral glucose metabolism pattern observed in our patient led to the differential diagnoses of PSP and neuroacanthocytosis which have rarely been discussed in the context of HD.^5^ PSP, FTD, and Alzheimer's disease all belong to the group of tauopathies.^24^ There is increasing evidence that HD might be molecularly defined as a tauopathy as well.^25^ This assumption is based on the observation that intraneuronal tau protein inclusions and altered huntingtin protein influence the splicing process of tau messenger ribonucleic acid (mRNA).^25^, ^26^

## 4 CONCLUSION

The presented case adds to the body of evidence suggesting a clinical and morphological overlap between HD and tauopathies.

As novel disease‐modifying treatments for HD will soon become clinically relevant, early diagnosis of HD will become increasingly important in order to allow a causal and molecularly tailored therapy. There are promising clinical trials with antisense oligonucleotides which inhibit the overexpression of the *HTT* gene in HD patients, suggesting that an early treatment initiation in mildly affected or even presymptomatic cases might yield the greatest therapeutic benefit.^27^ To minimize the diagnostic delay, we suggest an interdisciplinary approach with special emphasis on a timely application of neuroimaging techniques, including ^18^F‐FDG PET/CT in particularly ambiguous cases.

## CONFLICTS OF INTERESTS

The authors have no conflicts of interest to declare.

## AUTHOR CONTRIBUTION

MSW, AO, and AG wrote the first draft of manuscript. JH commented on previous versions of the manuscript, language editing, and proofreading. NM contributed to the acquisition of MRI images. GB contributed to the acquisition and work up of ^18^F‐FDG PET/CT images. All authors read and approved the final manuscript.

## ETHICAL APPROVAL

Written informed consent for patient information to be published was provided by the patients’ legal guardian.

## Data Availability

The data that support the findings of this study are available from the corresponding author upon reasonable request.

## References

[1] McColgan P , Tabrizi SJ . Huntington's disease: a clinical review. Eur J Neurol. 2018;25(1):24‐34. 28817209 10.1111/ene.13413

[2] Ross CA , Tabrizi SJ . Huntington's disease: from molecular pathogenesis to clinical treatment. Lancet Neurol. 2011;10(1):83‐98. 21163446 10.1016/S1474-4422(10)70245-3

[3] Macdonald M . A novel gene containing a trinucleotide repeat that is expanded and unstable on huntington's disease chromosomes. the huntington's disease collaborative research group. Cell. 1993;72(6):971‐983. 8458085 10.1016/0092-8674(93)90585-e

[4] Anderson KE . Huntington's disease. Handb Clin Neurol. 2011;100:15‐24. 21496569 10.1016/B978-0-444-52014-2.00002-1

[5] Martino D , Stamelou M , Bhatia KP . The differential diagnosis of huntington's disease‐like syndromes: ‘Red flags’ for the clinician. J Neurol Neurosurg Psychiatry. 2013;84(6):650‐656. 22993450 10.1136/jnnp-2012-302532 PMC3646286

[6] Sutovsky S , Smolek T , Alafuzoff I , et al. Atypical huntington's disease with the clinical presentation of behavioural variant of frontotemporal dementia. J Neural Transm (Vienna). 2016;123(12):1423‐1433. 27287334 10.1007/s00702-016-1579-5

[7] Fernández‐Nogales M , Lucas JJ . Altered levels and isoforms of tau and nuclear membrane invaginations in huntington's disease. Front Cell Neurosci. 2020;13:574. 32009905 10.3389/fncel.2019.00574 PMC6978886

[8] McGowan Dp , van Roon‐Mom W , Holloway H , et al. Amyloid‐like inclusions in huntington's disease. Neuroscience. 2000;100(4):677‐680. 11036200 10.1016/s0306-4522(00)00391-2

[9] Paoli RA , Botturi A , Ciammola A , et al. Neuropsychiatric burden in huntington's disease. Brain Sci. 2017;7(6):67. 10.3390/brainsci7060067 PMC5483640 28621715

[10] Connors MH , Teixeira‐Pinto A , Loy CT . Psychosis and longitudinal outcomes in huntington disease: the COHORT study. J Neurol Neurosurg Psychiatry. 2020;91(1):15‐20. 31611263 10.1136/jnnp-2019-320646

[11] van Duijn E , Craufurd D , Hubers AA M , et al. Neuropsychiatric symptoms in a european huntington's disease cohort (REGISTRY). J Neurol Neurosurg Psychiatry. 2014;85(12):1411‐1418. 24828898 10.1136/jnnp-2013-307343

[12] Morris JC , Heyman A , Mohs RC , et al. The consortium to establish a registry for alzheimer's disease (CERAD). part I. clinical and neuropsychological assessment of alzheimer's disease. Neurology. 1989;39(9):1159‐1165. 2771064 10.1212/wnl.39.9.1159

[13] Berres M , Monsch AU , Bernasconi F , Thalmann B , Stähelin HB . Normal ranges of neuropsychological tests for the diagnosis of alzheimer's disease. Stud Health Technol Inform. 2000;77:195‐199. 11187541

[14] Benke T , Karner E , Delazer M . FAB‐D: German version of the frontal assessment battery. J Neurol. 2013;260(8):2066‐2072. 23649609 10.1007/s00415-013-6929-8

[15] Wang YP , Gorenstein C . Psychometric properties of the beck depression inventory‐II: a comprehensive review. Braz J Psychiatry. 2013;35(4):416‐431. 24402217 10.1590/1516-4446-2012-1048

[16] Ekselius L , Lindström E , von Knorring L , Bodlund O , Kullgren G . SCID II interviews and the SCID screen questionnaire as diagnostic tools for personality disorders in DSM‐III‐R. Acta Psychiatr Scand. 1994;90(2):120‐123. 7976457 10.1111/j.1600-0447.1994.tb01566.x

[17] Duff K , Paulsen JS , Beglinger LJ , Langbehn DR , Stout JC , Predict‐HD Investigators of the Huntington Study Group . Psychiatric symptoms in huntington's disease before diagnosis: the predict‐HD study. Biol Psychiatry. 2007;62(12):1341‐1346. 17481592 10.1016/j.biopsych.2006.11.034

[18] Nagel M , Rumpf HJ , Kasten M . Acute psychosis in a verified huntington disease gene carrier with subtle motor signs: psychiatric criteria should be considered for the diagnosis. Gen Hosp Psychiatry. 2014;36(3):361.e3‐361.e4. 10.1016/j.genhosppsych.2014.01.008 24576988

[19] Ding J , Gadit AM . Psychosis with huntington's disease: role of antipsychotic medications. BMJ Case Rep. 2014;2014:bcr2013202625. 10.1136/bcr-2013-202625 PMC4139575 25139915

[20] Corrêa BB , Xavier M , Guimarães J . Association of huntington's disease and schizophrenia‐like psychosis in a huntington's disease pedigree. Clin Pract Epidemiol Ment Health. 2006;2:1. 16480508 10.1186/1745-0179-2-1 PMC1386660

[21] Nielsen TR , Bruhn P , Nielsen JE , Hjermind LE . Behavioral variant of frontotemporal dementia mimicking huntington's disease. Int Psychogeriatr. 2010;22(4):674‐677. 20170589 10.1017/S1041610210000098

[22] Roussakis AA , Piccini P . PET imaging in huntington's disease. J Huntingtons Dis. 2015;4(4):287‐296. 26683130 10.3233/JHD-150171 PMC4927896

[23] Pagano G , Niccolini F , Politis M . Current status of PET imaging in huntington's disease. Eur J Nucl Med Mol Imaging. 2016;43(6):1171‐1182. 26899245 10.1007/s00259-016-3324-6 PMC4844650

[24] Ludolph AC , Kassubek J , Landwehrmeyer BG , et al. Tauopathies with parkinsonism: clinical spectrum, neuropathologic basis, biological markers, and treatment options. Eur J Neurol. 2009;16(3):297‐309. 19364361 10.1111/j.1468-1331.2008.02513.x PMC2847416

[25] Gratuze M , Cisbani G , Cicchetti F , Planel E . Is huntington's disease a tauopathy? Brain. 2016;139(Pt 4):1014‐1025. 26969684 10.1093/brain/aww021

[26] Vuono R , Winder‐Rhodes S , de Silva R , et al. The role of tau in the pathological process and clinical expression of huntington's disease. Brain. 2015;138(Pt 7):1907‐1918. 25953777 10.1093/brain/awv107 PMC4572485

[27] Tabrizi SJ , Leavitt BR , Landwehrmeyer GB . Targeting huntingtin expression in patients with huntington's disease. N Engl J Med. 2019;381(14):1398. 31577898 10.1056/NEJMx190024

